# Citric Acid Capped CdS Quantum Dots for Fluorescence Detection of Copper Ions (II) in Aqueous Solution

**DOI:** 10.3390/nano9010032

**Published:** 2018-12-27

**Authors:** Zhezhe Wang, Xuechun Xiao, Tong Zou, Yue Yang, Xinxin Xing, Rongjun Zhao, Zidong Wang, Yude Wang

**Affiliations:** 1 School of Materials Science and Engineering, Yunnan University, Kunming 650091, China; wzz0307@yeah.net (Z.W.); zoutong626@163.com (T.Z.); rjzhao0504@hotmail.com (R.Z.); WangZiDongK@163.com (Z.W.); 2 Department of Physics, Yunnan University, Kunming 650091, China; Yangyue_018@163.com (Y.Y.); xingxinxin@126.com (X.X.); 3 Key Lab of Quantum Information of Yunnan Province, Yunnan University, Kunming 650091, China

**Keywords:** fluorescence sensor, citric acid, CdS quantum dots, copper ions, high selectivity

## Abstract

Citric acid capped CdS quantum dots (CA-CdS QDs), a new assembled fluorescent probe for copper ions (Cu^2+^), was synthesized successfully by a simple hydrothermal method. In this work, the fluorescence sensor for the detection of heavy and transition metal (HTM) ions has been extensively studied in aqueous solution. The results of the present study indicate that the obtained CA-CdS QDs could detect Cu^2+^ with high sensitivity and selectivity. It found that the existence of Cu^2+^ has a significant fluorescence quenching with a large red shifted (from greenish-yellow to yellowish-orange), but not in the presence of 17 other HTM ions. As a result, Cu_2_S, the energy level below the CdS conduction band, could be formed at the surface of the CA-CdS QDs and leads to the quenching of fluorescence of CA-CdS QDs. Under optimal conditions, the copper ions detection range using the synthesized fluorescence sensor was 1.0 × 10^‒8^ M to 5.0 × 10^‒5^ M and the limit of detection (LOD) is 9.2 × 10^‒9^ M. Besides, the as-synthesized CA-CdS QDs sensor exhibited good selectivity toward Cu^2+^ relative to other common metal ions. Thus, the CA-CdS QDs has potential applications for detecting Cu^2+^ in real water samples.

## 1. Introduction

Copper, a cofactor in many important enzymes, is one of the essential trace elements for the various pathological and physiological activities in the human body [1,2,3]. Copper is also a vitally important transition metal which is extensively applied to a lot of respects to industrial and agricultural produce [4,5]. Despite the deficiency in copper in the body potentially resulting in a variety of health problems [6,7], excess copper can cause various health problems such as eczema, kidney disease, gastrointestinal diseases, and damage to serious nervous diseases [8,9,10,11,12,13,14]. The high concentration of copper ions on the water can also cause great influence on aquatic organisms. What’s more, copper pollution has become a serious environmental issue due to the discharge of industrial wastewater containing copper (II) ions to surface water [15]. Therefore, China has stipulated that the maximum level of Cu^2+^ is 1 mg L^−1^ (15.7 μM) according to standard GB3838-2002 and GB5749-2006 [16,17]. Currently, the U.S. Environmental Protection Agency (EPA) action level for copper in drinking water is 20.41 μM [18,19]. Therefore, detecting Cu^2+^ with high sensitivity and selectivity possesses important meaning for the field of occupational safety and human health.

Until now, great efforts have been devoted to the development of techniques for the determination of copper (II) in water, such as atomic absorption spectrometry (AAS) [20,21], inductively coupled plasma-mass spectroscopy (ICP-MS) [22,23], inductively coupled plasma optical emission spectrometry (ICP-OES) [24,25], anodic cathodic stripping voltammetry (ACSV) [26], electrochemical [27] etc. However, there are disadvantages of these measuring methods such as being time-consuming, having complicated procedures and expensive instruments. Thus, simple and rapid testing methods for the detection of copper ions in an aqueous solution are an urgent need. In recent years, the fluorescence techniques have received considerable attention owing to their advantages such as feasibilities, cost-effectiveness, and rapid procedures [28].

Nowadays, semiconductor quantum dots (QDs), contrasting organic fluorophores, have many obvious advantages of high photobleaching threshold, high fluorescence intensity, narrow excitation, broad emission bands and excellent photostability [29,30,31,32]. Therefore, semiconductor QDs have great potential applications for solar cells [33,34], light-emitting diodes [35], and ultraviolet absorbers [36,37]. Moreover, QDs as a fluorescence sensor have been extensively studied in determination of copper ions [2,38,39,40]. These preponderances of optical properties lead to the increasing use of quantum dots as the ideal fluorescent probes for the determination of metal ions [41]. Besides, semiconductor QDs can be further modified by surface ligands and therefore obtain specific fluorescent prober. So far, these surface ligands with functional groups, including citrate [42], L-cysteine [43,44], cysteamine [45,46], 3-mercaptopropionic acid [47], thioglycolic acid [48], glutathione [49] and peptide [50], which have been studied in CdS QDs modification and analytical application. Therefore, it could be expected that surface ligands modified CdS QDs as fluorescence probe for detecting Cu^2+^ show promise.

Herein, water soluble citric acid capped CdS QDs was synthesized by a simple one-pot hydrothermal method of cadmium chloride hemi(pentahydrate), thioacetamide and citric acid. The obtained CdS QDs shows a high sensitivity and selectivity based on fluorescence quenching of QDs caused by the interactions between QDs surface and Cu^2+^. The fluorescence intensity was reduced and a red-shift in emission was observed in the presence of Cu^2+^. The synthesized CA-CdS QDs fluorescent probe has been found to response only with Cu^2+^ and makes their detection easy about the presence of other interfering metal ions, such as K^+^, Ca^2+^, Mg^2+^, Al^3+^, Fe^2+^, Fe^3+^, Mn^2+^, Co^2+^, Cr^3+^, Cd^2+^, Na^+^, Ni^2+^, Zn^2+^, Pb^2+^, Hg^2+^, Li^+^ and La^3+^. The obtained CA-CdS QDs as fluorescence sensor shows excellent selectivity and sensitivity in the detection of Cu^2+^.

## 2. Materials and Methods

### 2.1. Materials

Cadmium chloride hemi(pentahydrate) (CdCl_2_·2½H_2_O, Chengdu Kelon Chemical Reagent Factory, Chengdu, China), thioacetamide (TAA, Tianjin Zhiyuan Chemical Reagent Co. Ltd., Tianjin, China), Citric acid (C_6_H_8_O_7_, Tianjin Zhiyuan Chemical Reagent Co. Ltd., Tianjin, China), sodium hydroxide (NaOH, Aladdin, Shanghai, China), trihydroxymethylaminomethane (Tris, Macklin, Shanghai, China), hydrochloric acid (Chengdu Kelon Chemical Reagent Factory, Chengdu, China) and all the metal salts were purchased from local supplier as guaranteed-grade reagents, without any additional purification. The different aqueous solution prepared to use distilled water. The chemical reagent used in the experiment was listed in Table S1.

### 2.2. Instrumentations

The transmission electron microscopy (TEM) and high-resolution transmission electron microscopy (HRTEM) images of the CA-CdS QDs were carried out on a JEM-2100 Electron Microscopy (200 kV, JEOL, Tokyo, Japan). The SPA-400 SPM atomic force microscope (AFM, JEOL, Tokyo, Japan) was applied to surface topography analysis of the sample. X-ray diffraction (XRD, Rigaku D/MAX-3B, Rigaku, Tokyo, Japan) with a Cu Kα radiation (λ = 1.54056 Å) was used for the crystalline structure of the CA-CdS QDs. The thermal gravimetric analysis curves (TGA) were obtained on an American TA SDT-2960 thermal analyzer (TA, New Castle, DE, USA). The element distribution of the sample was tested on Hitachi S-4800 field emission electron microscopy (FESEM, Hitachi, Japan) with the energy dispersive X-ray (EDX) device. X-ray photoelectron spectroscopy (XPS) was analyzed by a K-Alpha system (Thermo fisher, New York, NY, USA) at room temperature. The fluorescence spectra were measured by the Horiba Fluorolog-3 spectrofluorometer (Horiba, New York, NY, USA) with an emission slit width set at 3 nm. The fluorescence quantum yield was obtained according to the following equation [14]:

QY(sample) = (*F*_sample_/*F*_ref_)(*A*_ref_/*A*_sample_)(*η*_sample_^2^/*η*_ref_^2^)QY_ref_ (1)

*F*, *A* and *η* are the spectrally integrated photon fluxes (area under the emission spectra), the absorbance at the excitation wavelength and the refractive index of the solvent, respectively. Rhodamine 6G in ethanol as the reference standard (QY = 95%). Hamamatsu compact fluorescence lifetime spectrometer C11367 (Quantaurus-Tau, Hamamatsu, Japan), using an LED light source with *λ*_ex_ = 365 nm, was used to measure the time resolved photoluminescence lifetime decays. Fourier transformed infrared (FTIR) spectra were performed on an AVATAR360 FT-IR spectrophotometer (Thermo Fisher, New York, NY, USA) during the range 400–4000 cm^‒1^. Raman spectra were measured at room temperature using a Renishaw inVia Raman microscope (Renishaw, London, UK). UV-vis spectra were recorded with a UV-1800 spectrophotometer from Jinghua Instruments (Shanghai, China) with a wavelength range between 260 nm and 800 nm. An inoLab pH Level 1 (Weilheim, Germany) precision pH meter was used for testing the pH value.

### 2.3. Methods

The CdS QDs was synthesized using citric acid as the surface modification through hydrothermal method [42,48]. In brief, 0.25 mmol CdCl_2_·2.5H_2_O and 0.5 mmol citric acid were dissolved in 50 mL distilled water under magnetic stirring. After 15 min, 1 M NaOH, as a pH regulator, adjust pH values to 10. Then, 0.0625 mmol TAA was added under magnetic stirring. With another magnetic stirring for 30 min, the mixture solution was transferred to a 100-mL Teflon-lined stainless-steel autoclave, which was maintained at 120 °C for 2 h and then cooled to room temperature. Finally, a bright yellow solution was obtained. The obtained solution was transferred to dialyze using a dialysis bag with a cutoff molecular weight of 3500 Da for 4 h. The synthesized CA-CdS QDs solution was washed with ethanol and water by centrifugation (8000 rpm) and dried at 40 °C for 24 h, a yellow powder was obtained. The yellow powder was characterized by FTIR, Raman and XRD spectra. The Mapping and EDS were measured by filter which was soaked in CA-CdS QDs solution

### 2.4. Selective Detection of Cu^2+^

To study the sensitivity and selectivity of the proposed system, the following procedure was carried out. The pH value of synthesized CA-CdS QDs solution was regulated by 0.3 M Tris and 0.3 M HCl buffer solution. After the contrast experiments, the maximum fluorescence quenching was obtained at pH 8.0. The different concentrations of the aqueous solution for all metal ions were prepared using distilled water and stored at room temperature. The various concentrations of metal ion were prepared by mixing the required amount of metal ion solution with CA-CdS QDs solution. The concentration of interference metal ions was 50 μΜ. Initially, 0.5 mL 0.3 M Tris buffer solution and 0.25 mL 0.3 M HCl buffer solution was added into a 10 mL calibrated test tube. The solution was diluted with ultrapure water to 5 mL. Then 0.5 mL CA-CdS QDs and 100 μL various concentrations of Cu^2+^ were added into the mixture solution. The fluorescence intensity was measured after equilibrating for 20 min.

## 3. Results and Discussion

### 3.1. Characterization

**Figure 1:**
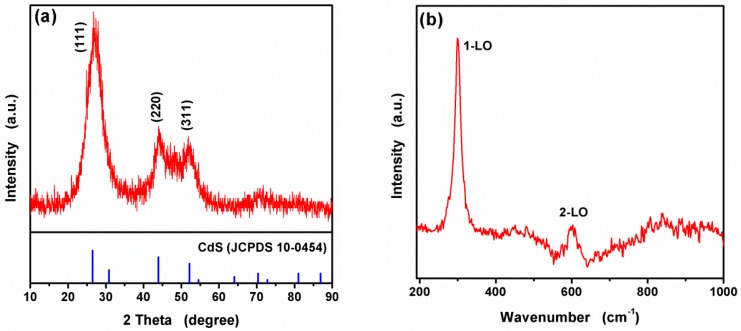
X-ray diffraction pattern (**a**) and Raman spectra (**b**) of as-synthesized CA-CdS QDs.

The compositions and crystalline structures of the obtained CA-CdS were characterized as follows. The X-ray diffraction pattern of the synthesized CA-CdS QDs was shown in Figure 1a. The peaks of the pattern indicated the as-synthesized CA-CdS QDs is crystalline and the presence of (111), (220), and (311) planes agrees well with the cubic zinc blende structure of CdS (JCPDS card no. 10-0454). The widened XRD diffraction peaks indicate that the synthesized CA-CdS QDs has a small size. The crystallite sizes were estimated to be 8.2 nm according to Scherrer equation. The nanoparticles were extracted by adding 0.1 M HCl from this solution. Perhaps, the citric acid ligand adsorbs weakly onto the CA-CdS QDs surfaces under the acidic condition, resulting in the CA-CdS QDs lead to rapid aggregation and attributing the structure to the sample. The Raman spectra of the synthesized CA-CdS QDs carried presented in Figure 1b. Two prominent peaks at ~300 and ~600 cm^−1^ represent a well agreement with the first-order longitudinal optical (1LO) phonon peak and the second-order longitudinal optical (2LO) phonon peak of CdS [51], respectively. These results correspond to the Raman spectral data reported on the literature [52,53].

**Figure 2:**
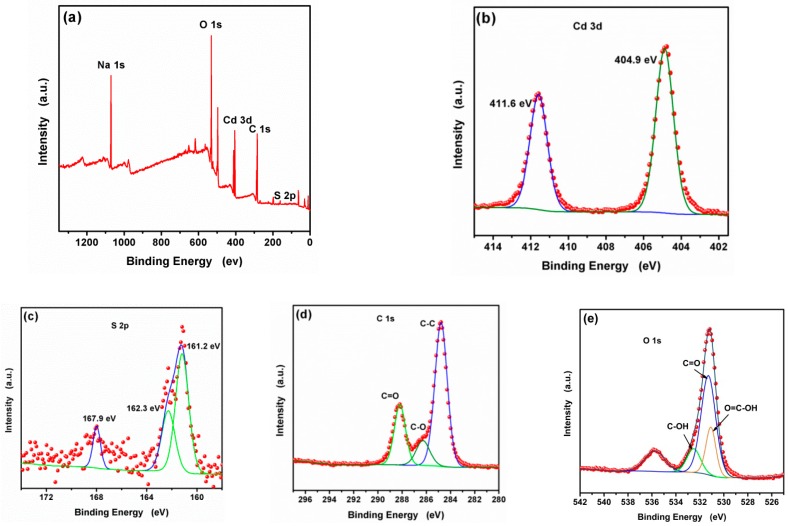
The (**a**) survey, (**b**) Cd 3d, (**c**) S 2p, (**d**) C 1s and (**e**) O 1s XPS spectra of the CA-CdS QDs.

The XPS spectra were analyzed to investigate the composition and chemical states of the as-synthesized CA-CdS QDs. The resulting survey spectra are presented in Figure 2. The XPS spectra of O 1s, Cd 3d, S 2p, C 1s, and Na 1s were displayed in Figure 2a, which indicates the existence of Cd, S, C and O elements. Figure 2b displays the peaks at 404.9 eV and 411.6 eV are in agreeance with Cd 3d_5/2_ and Cd 3d_3/2_ peaks, respectively. In Figure 2c, the characteristic peaks centering at 161.2 eV and 162.3 eV correspond to S 2p_3/2_ and S 2p_1/2_, respectively. The peak at 167.9 eV was assigned to the form of SO_2_. As depicted in Figure 2d, the C 1s peaks show their binding energy values at 284.8 eV, 286.3 eV and 288.2 eV, which are ascribed to C–H/C–C, C–OH, and C(=O)O, respectively [54]. The O 1 s spectra Figure 2e indicates three peaks with binding energies at 535.8 eV (Auguer peak of sodium), 532.8 eV (–C–O–H) and 531.26 eV (–O–C=O), respectively. A simultaneous TGA and DTG analysis of synthesized CA-CdS QDs by hydrothermal method without calcination at a heating rate of 10 °C/min from room temperature to 800 °C in N_2_. The result was shown in Figure S1. The boiling point of citric acid is approximately 470 °C. The molar ratio of the citric acid ligand to CdS is about 1:9. This illustrates that the presence of hydroxyl and carboxylic groups on the surface and citric acid successfully decorated on the surface of CdS QDs [54].

**Figure 3:**
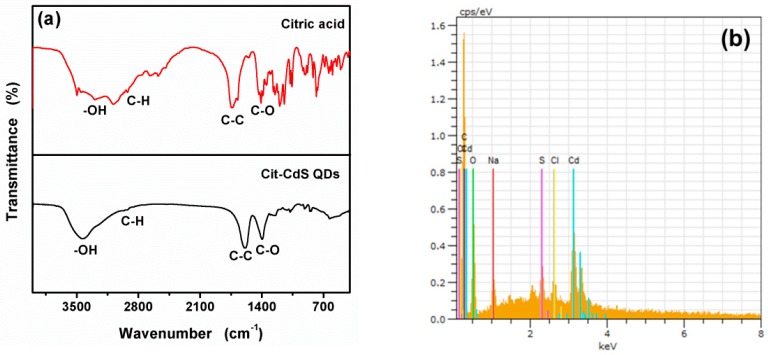

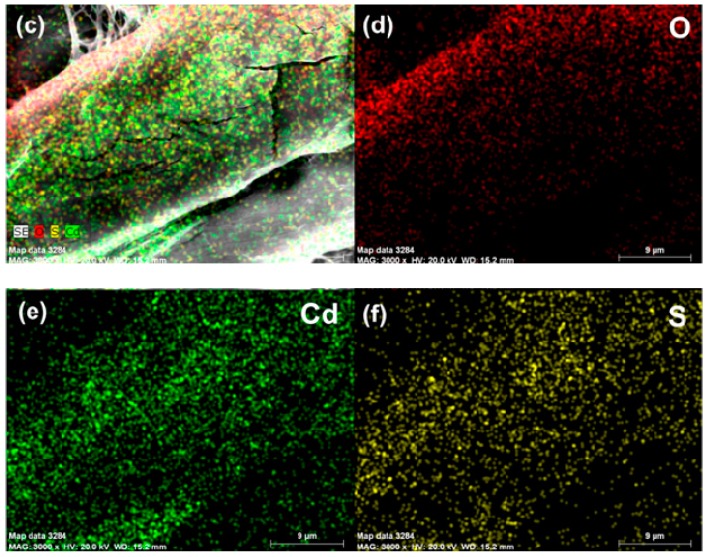
FTIR spectra (**a**), EDX analysis (**b**) and elemental mapping (**c**–**f**) of the prepared CA- CdS QDs.

To further confirm the citric acid decorated on the surface of CdS QDs, Figure 3 shows the FT-IR spectra of citric acid and citric acid modified CdS QDs. The absorption at 2923 cm^−1^ was due to the C–H stretching of citric acid and broad peak appeared at 3000–3600 cm^−1^ corresponded to the absorption of hydrogen bonded O–H groups in citric acid. The characteristic peaks of C–C stretching and C–O asymmetrical appear at 1741 cm^−1^, and 1471 cm^−1^, respectively [55]. The citric acid modified CdS QDs showed similar FT-IR spectra with three characteristic peaks. The peaks located at 1395 and 2935 cm^−1^ match well with the C–H bending. The characteristic peaks of C=O stretching and C–O asymmetrical appear at 1251, and 1588 cm^−1^, respectively [56]. The results suggest that citric acid successfully decorated the surface of CdS QDs. The EDX spectrum (Figure 3b) further confirms the CdS QDs has been successfully modified by citric acid, because C and O elements are attributed to citric acid. As shown in Figure 3c–f), the element mapping of as-synthesized CA-CdS QDs was carried out for analyzing the element distribution. The results indicated that uniform distribution of Cd, S and O throughout was displayed.

**Figure 4:**
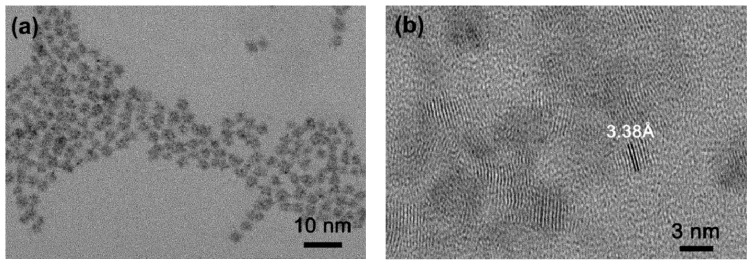

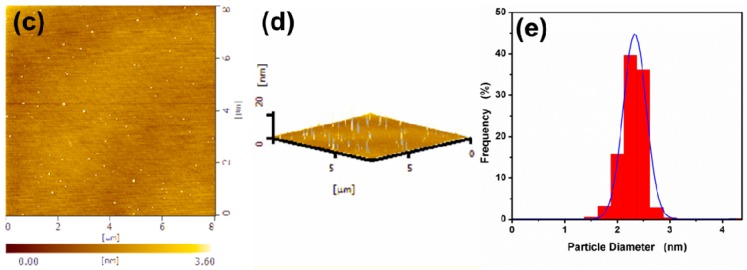
TEM and HRTEM images (**a**,**b**) of the as-synthesized CA-CdS QDs; (**c**) and (**d**) represents 2D and 3D AFM images of CA-CdS QDs deposited on silicon substrate, respectively; (**e**) Statistical analysis of the heights of CA-CdS QDs measured by AFM.

The size, shape and morphologic of CA-CdS QDs were studied by the TEM, HRTEM and AFM images (Figure 4). It was found that these particles are quasi-spherical and are uniform, which were approximately 2.5 nm in diameter. The HRTEM image (Figure 4b) indicates a lattice spacing of 3.38 Å, which corresponds to the (111) plane of the cubic zine blende structure of CdS. AFM was used to examine further the morphology of synthesized CA-CdS QDs. The typical two-dimensional (2D), three-dimensional (3D) AFM images were shown in Figure 4c,d, respectively. According to the result, the size of CA-CdS QDs was recorded. Figure 4e exhibited the histogram of the height of the CA-CdS QDs, which shows mostly QDs are 2.3 ± 0.5 nm sized.

**Figure 5:**
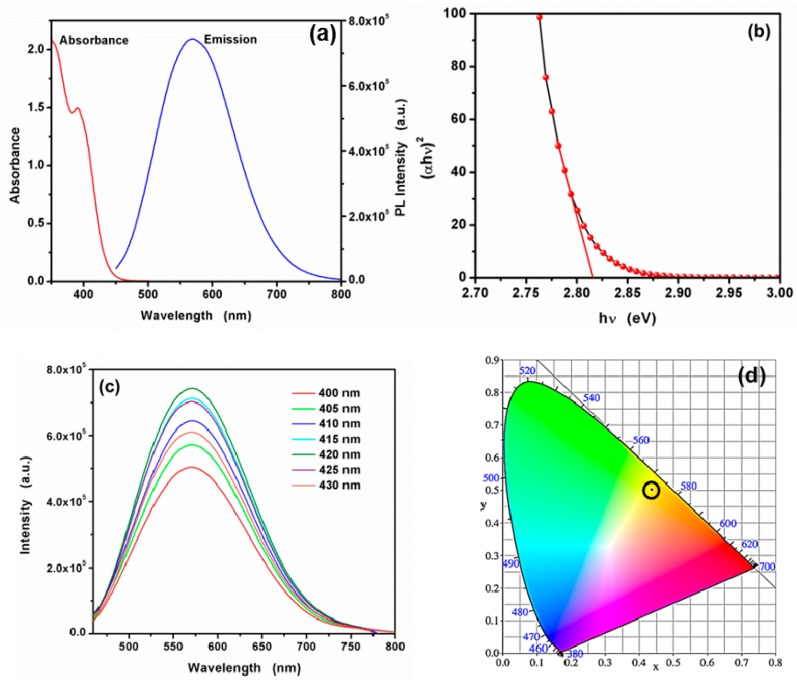
(**a**) The UV-vis absorbance (red line), the emission spectra (blue line) of the as-synthesized CA-CdS QDs. (**b**) (*αhυ*)^2^*vs*. *hυ* plot of CdS QDs for the determination of band gap. (**c**) The excitation-dependent emission spectra of CA-CdS QDs and (**d**) The CIE 1931 coordinates.

The absorption spectra (Figure 5a) exhibits the absorption edges of the CA-CdS QDs, showing a large blue shift compared with the bulk CdS absorption peak at 515 nm [57]. Generally, the blue shift of absorption peak was caused by quantum confinement [58]. The absorption edge, obtained by the intersection of the sharply decreasing region of the spectrum with the baseline, corresponds to band gap of 2.84 eV [47]. As shown in Figure 5b, according to the linear between (*αhυ*)^2^ and photon energy (*hυ*) [59,60,61], the band energy was determined to be 2.81 eV. In contrast of the band gaps of CA-CdS QDs by the two methods mentioned above, the same result could be concluded. Based on the effective mass approximation, the Brus equation was applied for determining the grain size of CA-CdS QDs [61,62,63,64,65]:

*E*(QDs) = *Eg* + (*h*^2^/8*μR*^2^) − (1.8*e*^2^/4*πεε*_0_*R*) (2)

where *Eg* is the band gap of the bulk CdS (2.42 eV), h is the reduced Plank’s constant (6.625 × 10^−34^ J·s), *R* is the radius of particle, e is the charge of the electron (1.6 × 10^−19^ C), *ε*_0_ is the vacuum dielectric constant (*ε*_0_ = 8.854 × 10^−12^ F/m), *ε* is the semiconductor dielectric constant (5.7), *μ* = (1/*m*_e_ + 1/*m*_h_)^−1^, *m*_e_ and *m*_h_ are effective masses of the electrons and holes, respectively, and *m*_0_ is the free electron mass (*m*_0_ = 9.108 × 10^−31^ kg). With the effective masses of electrons (*m*_e_ = 0.19 *m*_0_) and holes (*m*_h_ = 0.8 *m*_0_) the grain size of synthesized CA-CdS QDs was calculated to be 2.01 nm, which is consistent with the AFM and HRTEM. A large Stokes shift was observed, which is ascribed to be due to the surface defect emission [49]. The emission spectrum (Figure 5c) manifests that the emission peak remains little changed with the variation of excitation wavelength in the range of 400–430 nm. The result suggests that the maximum emission spectrum is obtained at an excitation wavelength of 420 nm. The CA-CdS QDs emitted greenish yellow color was exhibited on the corresponding CIE 1931 chromaticity diagram (Figure 5d) with a corresponding CIE coordinates at (0.44, 0.50) and the correlated color temperature (CCT) is 3652.7 K. The quantum yield of the CA-CdS QDs was calculated to be 18.82% when the original solution was diluted 200 times. Thus, the CA-CdS QDs will have good application prospects in analytical chemistry.

### 3.2. Selective Fluorescence Quenching of CA-CdS QDs by Cu^2+^

Generally, the selectivity is a pivotal parameter for the fluorescence sensors. The fluorescence intensity of the sensors should be affected only by the target ions without interfering with other metal ions, which is still a difficult subject due to many metal ions having potential effects on the QDs fluorescence intensity. To assess the selectivity of the proposed fluorescence sensors system, the influences of various coexistence ions on the fluorescence intensity of CA-CdS QDs system were studied. In this work, the response to other commonly metal ions such as Na^+^, Ba^2+^, K^+^, Al^3+^, Mg^2+^, Cr^3+^, Hg^2+^, Mn^2+^, Cd^2+^, Co^2+^, Cu^2+^, Pb^2+^, Ca^2+^, Fe^3+^, Fe^2+^, La^3+^, Zn^2+^ and Ni^2+^ ions at a concentration of 50 μM were monitored in aqueous solutions, respectively.

**Figure 6:**
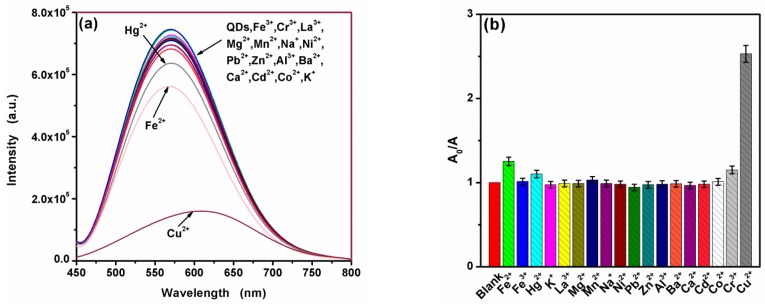
(**a**) Effect of various metal ions to fluorescence spectra of CA-CdS QDs and (**b**) relative fluorescence area (A_0_/A) of CdS QDS with various metal ions at a concentration of 50 µM.

As shown in Figure 6a, a fluorescence quenching was observed with the addition of Cu^2+^, while a large red shift appeared. The fluorescence area was used to check the quenching considering the important red shift. Besides, contrasting the fluorescence area of CA-CdS QDs before and after add interfering ions (Figure 6b), it can be easily observed that a significant fluorescence quenching upon addition of Cu^2+^. Hg^2+^ and Cr^3+^ ions have slightly quenched the fluorescence area. These results suggested that only Cu^2+^ causes a measurable fluorescence quenching with a large red shift to CA-CdS QDs. The pure citric acid was handled through hydrothermal using the same process as preparing the CA-CdS QDs, in order to eliminate the effects of citric acid. As shown in Figure S2a, the possibility for citric acid decomposes into carbon dots is minimal, because of the undetectable fluorescence intensity compared with CA-CdS QDs. The fluorescence spectrum of ultrapure water, citric acid solution after the heat treatment in the absence and in the presence of Cu^2+^ are shown in Figure S2b. The result indicates that the fluorescent center in citrate acid is caused by the back of the water. Hence, Citric acid has no effect on the fluorescence detection system and the CA-CdS QDs can be applied as a selective fluorescence sensor for Cu^2+^ by the fluorescence quenching.

### 3.3. Mechanism for the Interaction Between Cu^2+^ and CA-CdS QDs

The existence of Cu^2+^ leads to the fluorescence quenching of CA-CdS QDs in solution. Red shift appears on the band-edge emission from the fluorescence spectrum. The red shift can be considered that a new energy level below the conduction band (CB) of CdS was formed due to photochemical reduction of some pre-adsorbed Cu^2+^ by the CA-CdS QDs [60]. The XPS spectra of elements in the CA-CdS QDs after the interaction with Cu^2+^ are presented in Figure S3. According to the Figure S3f, the Cu 2p_3/2_ peaks shown at 932 and 933.1 eV can be assigned to the Cu^+^ and Cu^2+^ states, respectively. The peaks appearing at about 952 and 954.1 eV are ascribed to the existence of Cu^+^ and Cu^2+^, respectively. As a result, Cu_2_S exists on the surface of CA-CdS QDs. The lower solubility products (*K*_sp_) of either CuS or Cu_2_S than that of CdS (the Ksp of CuS, Cu_2_S, and CdS are 8 × 10^−37^, 3 × 10^−49^ and 1 × 10^−27^ respectively) [60]. Thus, it can be guessed that Cu^+^ and Cu^2+^ could precipitate out of the solution and sulfides deposit at the CA-CdS QDs surface. Moreover, the fluorescence spectra of CA-CdS QDs showed substantial red shift upon addition of Cu^2+^. It can be concluded the exclusive Cu_2_S deposition at the surface of CA-CdS QDs.

**Figure 7:**
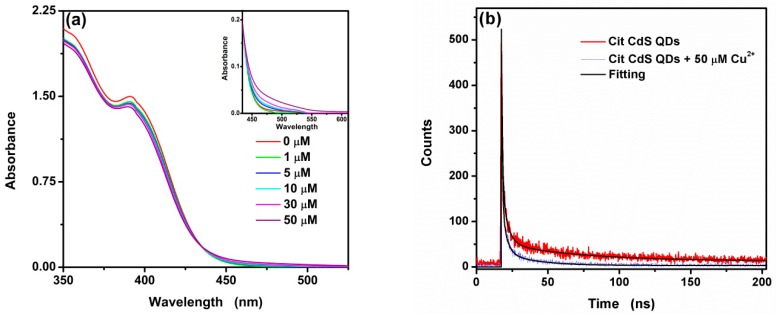
UV–vis spectra (**a**) and fluorescence lifetime decays (**b**) of CA-CdS QDs with different Cu^2+^ concentration.

In order to verify the proposes of the exclusive Cu_2_S deposition at the surface of CA-CdS QDs, the UV–vis spectra of CA-CdS QDs with 0, 1, 5, 10, 30 and 50 μM were measured, respectively, and displayed in Figure 7a. The absorbance peak is unchanged at all of the absorbance spectra at 390 nm while the new absorption peak arises at 525 nm. The results suggest that the change of absorption spectra is not attributed to the growth of large CA-CdS particles due to the absorption of bulk CdS at 515 nm, which further confirms Cu^2+^ bind onto the surface of CA-CdS QDs and get reduced to Cu^+^ upon addition of Cu^2+^. In order to improve understanding the interaction between CA-CdS QDs and Cu^2+^, the fluorescence lifetime decay curves of CA-CdS QDs in the absence and presence of Cu^2+^ were measured. As shown in Figure 7b, it can be easily found that the fluorescence lifetime would consistently be shortened upon addition of Cu^2+^. All the decay lifetime curves can be fitted by a three-exponential formula [66,67,68] as:

*I* = *A*_1_*exp*(−t/*τ*_1_) + *A*_2_*exp*(−t/*τ*_2_) + *A*_3_*exp*(−t/*τ*_3_) + *c* (3)

where *τ*_1_, *τ*_2_ and *τ*_3_ are the components of the fluorescence lifetimes, respectively. *A*_1_, *A*_2_ and *A*_3_ are the corresponding fitting parameters, and *c* is a constant. These parameters of fitted values are shown in Table 1. The results suggest that the fluorescence lifetime τ dropped from 38.6 ns to 12.1 ns upon addition of Cu^2+^. It means that the electron-hole recombination process is accelerated due to the presence of Cu^2+^.

**Figure 8:**
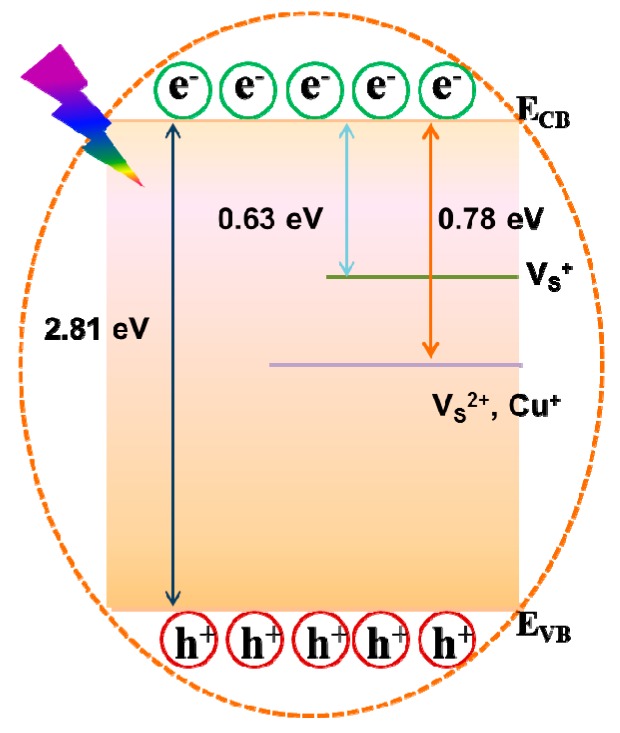
Energy-level diagram of Cu+ adsorbed on the surface of CA-CdS QDs.

The energy-level diagram of Cu^2+^ adsorbed on the surface of the CA-CdS QDs is displayed in Figure 8. Sulfur vacancies are easily formed into the surface of CA-CdS QDs. A great quantity of electrons will be excited from the valence band (VB) to the CB when the sample is under UV light. Meanwhile, photogenerated holes are produced at the VB. Subsequently, the mechanism of the coordination interaction between CA-CdS QDs and Cu^2+^ is as follows [69]:

In the absence of Cu^2+^
  (4) $$\mathrm{CdS}+h\upsilon_{\mathrm{ex}}\to \mathrm{CdS}(e^{-}/h^{+})\to \mathrm{CdS}(e_{\mathrm{tr}}^{-}/h_{\mathrm{tr}}^{+})$$
  (5) $$\mathrm{CdS}(e_{\mathrm{tr}}^{-}/h_{\mathrm{tr}}^{+})\to \mathrm{CdS}+h\upsilon_{1}(570\;\mathrm{nm})$$
In the presence of Cu^2+^
  (6) $$\mathrm{CdS}+h\upsilon_{\mathrm{ex}}\to \mathrm{CdS}(e^{-}/h^{+})+\mathrm{CdS}(e_{\mathrm{tr}}^{-}/h_{\mathrm{tr}}^{+})$$
  (7) $$\mathrm{CdS}(e_{\mathrm{tr}}^{-}/h_{\mathrm{tr}}^{+})+(V_{S}^{2+}\cdot {\mathrm{Cu}}^{+})\to \mathrm{CdS}(h_{\mathrm{tr}}^{+})+(V_{S}^{2+}\cdot {\mathrm{Cu}}^{+}e^{-})$$
  (8) $$\mathrm{CdS}(e_{\mathrm{tr}}^{-}/h_{\mathrm{tr}}^{+})+(V_{S}^{2+}\cdot {\mathrm{Cu}}^{+})\to \mathrm{CdS}+(V_{S}^{2+}\cdot {\mathrm{Cu}}^{+}e^{-})+\mathrm{heat}$$
  (9) $$\mathrm{CdS}(h_{\mathrm{tr}}^{+})+(V_{S}^{2+}\cdot {\mathrm{Cu}}^{+}e^{-})\to \mathrm{CdS}+(V_{S}^{2+}\cdot {\mathrm{Cu}}^{+})+h\upsilon_{2}(610\;\mathrm{nm})$$
**Process 4**: A great quantity of electrons will be excited from the VB to the CB when the sample is under UV light.
**Process 5**: The photogenerated electrons in the shallow or deep trap recombinated with the hole in the VB of CA-Cd QDs sent out yellow emission (~570 nm).
**Process 6**: The same process as the process 4.
**Process 7**: The ultra-small Cu_2_S are formed on the surface of CA-CdS QDs result in the presence of Cu^+^ sites. An electron was trapped by Cu^+^ sites.
**Process 8**: Recombination of shallow or deep trapped electrons in the CB with hole in the VB of CA-CdS QDs and some electron were trapped by Cu^2+^ sites with a new nonradiative channel.
**Process 9**: Recombination of shallow or deep trapped electrons in the CB with hole in the VB of CA-CdS QDs and sent out an orange emission (~610 nm).

Besides on these results, Cu^2+^ was absorbed at the surface of CA-CdS QDs and was reduced immediately to Cu^+^ at the low concentrations of Cu^2+^. Therefore, a new channel for electron-hole recombination was created because of the adsorbed Cu^2+^, both radiative and nonradiative. Thus, the electron-hole recombination process was accelerated and lead to reduced lifetime [70,71,72].

### 3.4. Effect of pH on the Fluorescence Quenching by Cu^2+^

**Figure 9:**
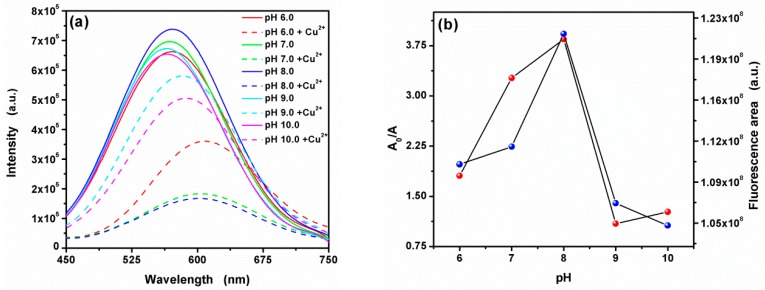
(**a**) Effect of pH on the fluorescence intensity and (**b**) Relative fluorescence area (A_0_/A) (red) and the pH dependence (blue)of CA-CdS QDs in the absence and presence of 50 µM Cu^2+^.

Basically, pH value acts an important role in the present fluorescence sensors system, which can influence the deprotonation or protonation process of the CA-CdS QDs [73]. In this work, the charge on the surface of the QDs would up to the degree of deprotonation of the carboxyl groups and may influence the electron-hole recombination process, thereby causing the fluorescence quenching. Thus, the pH value of this system was studied in the range of 6.0–10.0 with a 0.3 M Tris and 0.3 M HCl. As shown in Figure 9a, the fluorescence quenching is observed at all pH values. Besides, the fluorescence spectra show a large red shift due to the existence of Cu^2+^. As presented in Figure 9b. Maxima fluorescence area of the CA-CdS QDs was obtained at pH 8.0. The fact can be attributed to CA-CdS QDs was relatively more stable at pH 8.0 [42]. Citric acid dissociation will happen in the solution, if the H_3_A to represent the citric acid molecules and citric acid in the liquid phase is the H_3_A, H_2_A^−^, HA^2−^ and A^3−^ four forms coexist. When temperature is fixed, the form proportion is only affected by the pH value. The pKa1, pKa2 and pKa3 values of Citric acid were 3.13, 4.76 and 6.40, respectively. At low pH (pH < 6.0), the main existence of citric acid is H_2_A and HA^2−^. With pH value rises, the main formation of citric acid in solution was converted from HA^2−^ to A^3−^. When it comes to 8, the solution of CA-CdS QDs reaches its most stable state due to A^3−^ with Cd^2+^ complexing constant is greater than the H_2_A^-^ and HA^2-^. But if the pH is too high, a large number of OH combine with Cd^2+^ to form Cd(OH)_2_. As a result, the fluorescence intensity of quantum dots decreases. Therefore, at pH 8.0, the CA-CdS QDs provided the maximum fluorescence area and quenching area. Therefore, the pH 8.0 of sensing system was used in this work.

### 3.5. Sensitive Fluorescence Quenching of CA-CdS QDs by Cu^2+^

**Figure 10:**
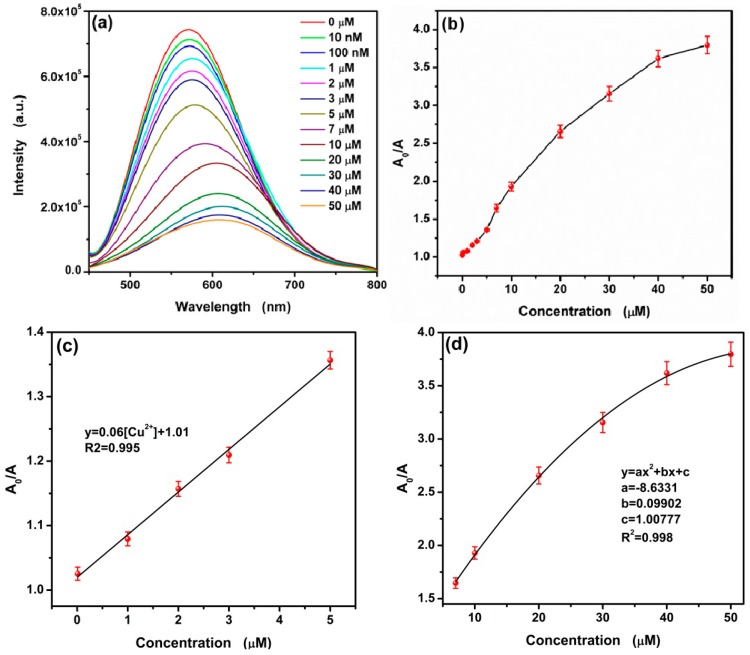
(**a**) Fluorescence spectra of CA-CdS QDs upon the addition of various concentrations of Cu^2+^, (**b**) the change between the relative fluorescence area (A_0_/A) of the CdS QDs and different levels of Cu^2+^, the calibration curve of Cu^2+^ detection in the range of 0–5μM (**c**) and 7–50 μM (**d**).

In order to evaluate the potential application of the CA-CdS QDs, the effects of various concentrations Cu^2+^ were investigated. The fluorescence intensity of CA-CdS QDs gradually quenching with the concentration increasing of Cu^2+^ has been shown in Figure 10a. The fluorescence quenching can be seen with a large red shift. As shown in Figure 10b,c, the calibration curve between A_0_/A (where A_0_ and A are the fluorescence area of the CA-CdS QDs in the absence and presence of Cu^2+^, respectively) and the concentration of Cu^2+^ are observed in the ranges of 0.01–5 μM and 7–50 μM, respectively. The correlative coefficient R^2^ is 0.995 and 0.998, respectively, indicating that the experimental data have a good agreement with the fitting curves. Based on a signal-to-noise ratio (S/N) of 3, the limit of detection (LOD) was calculated to be 9.2 nM.

**Figure 11:**
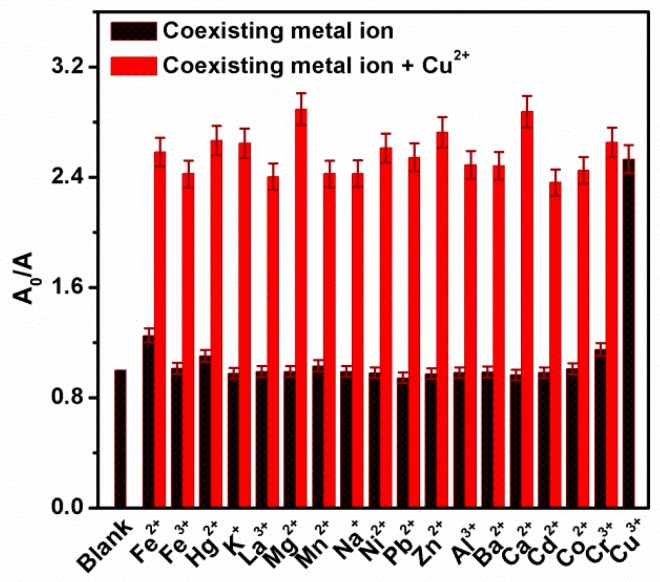
Plots of relative fluorescence area (A_0_/A) of CA-CdS QDs for various metal ions [M^n+^ = 50 μM]: the black bars display the response of individual metal ions while the red bars show the response of Cu^2+^ in the presence of other coexisting metal ions.

Compared with other fluorescence sensor in Cu^2+^ sensing performances shown in Table 2, the linear rang of this work is superior and outstanding LOD corresponding to the linear range of 0.01–5 μM is extremely low. In addition, the current system has better sensitivity. In order to study the anti-interference of the proposed system, the relative fluorescence area (A_0_/A) have been displayed (Figure 11). As a result, the sensing of Cu^2+^ is slightly influenced by the presence of generally coexisting ions. Therefore, the proposed sensing system has a potential application in detecting Cu^2+^ at low concentration levels with slightly influenced in the presence of interfering ions.

### 3.6. Application in Real Water Samples

In order to confirm the feasibility of the proposed sensors, Cu^2+^ ions in tap water as real water samples were studied. As Table 3 shows, the recoveries of Cu^2+^ ions were found to be above 95%. The results indicated that the satisfactory recoveries are obtained with relative standard deviation (RSD) less than 4.0%. The method is detecting successfully the real water samples, which attests the feasibility of the present sensor for the detection of Cu^2+^ ions with good accuracy and reliability. Therefore, the proposed QDs have potential applications for detecting Cu^2+^ ions as chemical and biological sensors.

## 4. Conclusions

In summary, citric acid capped CdS QDs has been synthesized through a simple hydrothermal method. A detailed characterization showed that citric acid successfully decorated on the surface of CA-CdS QDs. The CA-CdS QDs shows the excellent selectivity and sensitivity for Cu^2+^. The presence of Cu^2+^ leads to the fluorescence quenching of CA-QDs with a large red shift (from 570 to 610 nm). A linear relationship was obtained between the fluorescence response and the concentration of Cu^2+^ from 0.01 μM to 50 μM. The limit of detection (LOD) is 9.2 nM, which is far below the detection line of either EPA or Chinese standard. Moreover, the sensor exhibits good selectivity toward Cu^2+^ in the presence of other common metal ions. Therefore, the proposed sensor of CA-CdS QDs has the potential application in the determination of Cu^2+^.

## Figures and Tables

**Table 1:** The fitted values of the fluorescence lifetime decay curves.

Sample	*τ*	*A*_1_	*τ*_1_	*A*_2_	*τ*_2_	*A*_3_	*τ*_3_
CA-CdS QDs	38.6	432.267	0.711	137.739	3.958	39.992	53.070
CA-CdS QDs with Cu^2+^	12.1	541.551	0.552	118.948	3.272	30.542	22.198

**Table 2:** Comparison of different fluorescence sensor in Cu^2+^ sensing performances.

Fluorescent Probe	Linear range (μM)	Detection Limit (μM)	Sensitivity (μM)	Ref.
zinc-dopedAgInS_2_ QDs	0–340	0.0273	0.004	[2]
ZnSe QDs	1–6	0.47	0.22	[29]
PEG@ZnO QDs	0.01–0.2/2–10	0.0033	0.1768	[30]
CdTe-L QDs	0.515–15	0.015	0.4626	[31]
Cys-CdS QDs	2–10	1.5	0.001	[32]
CA-CdS QDs	0.01–5	0.0092	0.06	This work

**Table 3:** Analytical results of tap water samples.

Samples	Spiked (μM)	Found (μM)	Recovery (%, n = 5)	RSD (%, n = 5)
Tap water	10	9.97	99.7	2.9
	20	19.16	95.8	3.2

## Supplementary Materials

The following are available online at http://www.mdpi.com/2079-4991/9/1/32/s1, Table S1: Chemical reagent used in the experiment; Figure S1: TGA and DTG curves of as-synthesized CA-CdS QDs; Figure S2: (a) Fluorescence spectra of CdS QDs (red line) and citric acid solution (blue line). (b) Fluorescence spectra of H_2_O (khaki line), citric acid solution in the absence (red line) and in the presence (blue line) of Cu^2+^; Figure S3: The (a) survey, (b) Cd 3d, (c) S 2p, (d) C 1s, (e) O 1s and (f) Cu 2p XPS spectra for the CA-CdS QDs after interaction with Cu^2+^; Table S1: Chemical reagents used in the experiment.
