# Supplementary material for: Citric Acid Capped CdS Quantum Dots for Fluorescence Detection of Copper Ions (II) in Aqueous Solution
Source: Nanomaterials (Basel). 2018 Dec 27;9(1):32. doi: 10.3390/nano9010032 (PMC6358774; doi:10.3390/nano9010032)
Supplement: Supplementary file 1 [file nanomaterials-09-00032-s001.pdf]

Supplementary Materials

Table S1. Chemical reagent used in the experiment.

Chemical Formula	Purity	Producer	Country
$CdCl_2 \cdot 2\frac{1}{2}H_2O$	AR	Chengdu Kelon Chemical Reagent Factory	China
C_2H_5NS	AR	Tianjin Zhiyuan Chemical Reagent Co. Ltd.	China
NaOH	AR	Aladdin	China
$C_6H_8O_7 \cdot H_2O$	AR	Tianjin Zhiyuan Chemical Reagent Co. Ltd.	China
Tris	AR	Macklin	China
HCl	AR	Chengdu Kelon Chemical Reagent Factory	China
$FeCl_2 \cdot 4H_2O$	AR	Tianjin Zhiyuan Chemical Reagent Co. Ltd.	China
$FeCl_3 \cdot 6H_2O$	99.000%	Tianjin Fuchen Chemicals Reagent Factory	China
KCl	AR	Molbase	China
$LaCl_3$	AR	Tianjin Kemiou Chemical Reagent co., LTD	China
$Mg(NO_3)_2 \cdot H_2O$	AR	Tianjin Zhiyuan Chemical Reagent Co. Ltd.	China
$MnCl_2 \cdot 4H_2O$	99.0%	Tianjin Fuchen Chemicals Reagent Factory	China
NaCl	AR	Beijing Chemical Factory	China
$NiCl_2 \cdot 6H2O$	98.000%	Beijing Found Stat Science & Thechnology co., LTD	China
$Pb(NO_3)_2$	AR	Tianjin Zhiyuan Chemical Reagent Co. Ltd.	China
$ZnCl_2$	98.0%	Tianjin Fengchuan Chemical Reagent Technologles Co. Ltd.	China
$AlCl_3 \cdot 6H_2O$	AR	Tianjin Fengchuan Chemical Reagent Technologles Co. Ltd.	China
$BaCl_2 \cdot 2H_2O$	AR	Chengdu Kelon Chemical Reagent Factory	China
$CaCl_2$	96.00%	Tianjin Fengchuan Chemical Reagent Technologles Co. Ltd.	China
$CoCl_2 \cdot 6H_2O$	99.000%	Tianjin Fengchuan Chemical Reagent Technologles Co. Ltd.	China
$CrCl_3 \cdot 6H_2O$	AR	Tianjin Guangfu Fine Chemical Research Institute	China
$CuCl_2 \cdot 2H_2O$	99.0%	Tianjin Fengchuan Chemical Reagent Technologles Co. Ltd	China

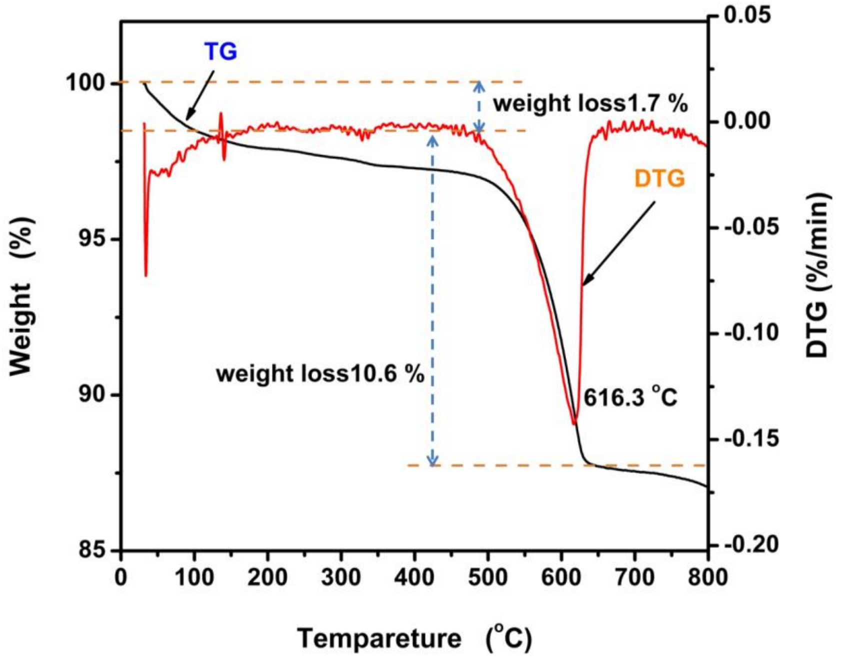

Figure S1. TGA and DTG curves of as-synthesized CA-CdS QDs.

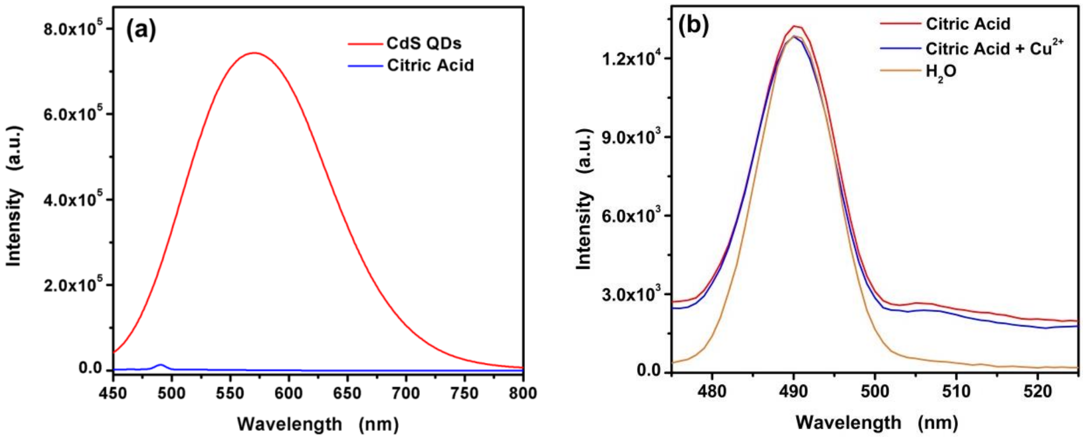

Figure S2. (**a**) Fluorescence spectra of CdS QDs (red line) and citric acid solution (blue line). (**b**) Fluorescence spectra of H_2O (khaki line), citric acid solution in the absence (red line) and in the presence (blue line) of Cu^{2+}.

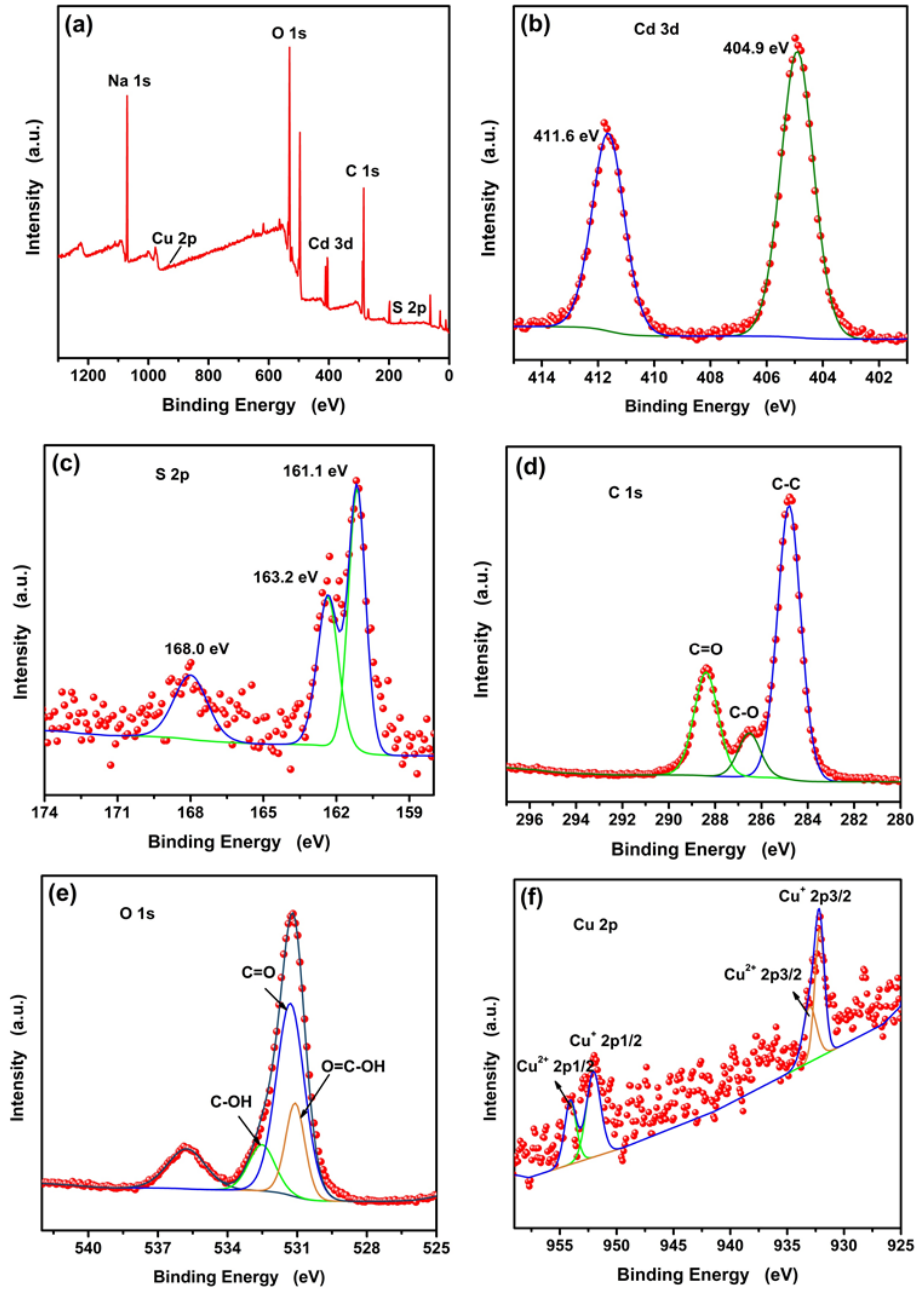

Figure S3. The (**a**) survey, (**b**) Cd 3d, (**c**) S 2p, (**d**) C 1s, (**e**) O 1s and (**f**) Cu 2p XPS spectra for the CA-CdS QDs after interaction with Cu^{2+}.
